# Gaze-action coupling, gaze-gesture coupling, and exogenous attraction of gaze in dyadic interactions

**DOI:** 10.3758/s13414-024-02978-4

**Published:** 2024-11-18

**Authors:** Roy S. Hessels, Peitong Li, Sofia Balali, Martin K. Teunisse, Ronald Poppe, Diederick C. Niehorster, Marcus Nyström, Jeroen S. Benjamins, Atsushi Senju, Albert A. Salah, Ignace T. C. Hooge

**Affiliations:** 1 Experimental Psychology, Helmholtz Institute, Utrecht University, Heidelberglaan 1, 3584CS Utrecht, Netherlands; 2 Information and Computing Sciences, Utrecht University, Utrecht, Netherlands; 3 Lund University Humanities Lab, Lund University, Lund, Sweden; 4 Department of Psychology, Lund University, Lund, Sweden; 5 Social, Health and Organisational Psychology, Utrecht University, Utrecht, Netherlands; 6 Research Center for Child Mental Development, Hamamatsu University School of Medicine, Hamamatsu, Japan

**Keywords:** Gaze, Eye tracking, Gestures, Action, Collaboration, Interaction

## Abstract

In human interactions, gaze may be used to acquire information for goal-directed actions, to acquire information related to the interacting partner’s actions, and in the context of multimodal communication. At present, there are no models of gaze behavior in the context of vision that adequately incorporate these three components. In this study, we aimed to uncover and quantify patterns of within-person gaze-action coupling, gaze-gesture and gaze-speech coupling, and coupling between one person’s gaze and another person’s manual actions, gestures, or speech (or exogenous attraction of gaze) during dyadic collaboration. We showed that in the context of a collaborative Lego Duplo-model copying task, within-person gaze-action coupling is strongest, followed by within-person gaze-gesture coupling, and coupling between gaze and another person’s actions. When trying to infer gaze location from one’s own manual actions, gestures, or speech or that of the other person, only one’s own manual actions were found to lead to better inference compared to a baseline model. The improvement in inferring gaze location was limited, contrary to what might be expected based on previous research. We suggest that inferring gaze location may be most effective for constrained tasks in which different manual actions follow in a quick sequence, while gaze-gesture and gaze-speech coupling may be stronger in unconstrained conversational settings or when the collaboration requires more negotiation. Our findings may serve as an empirical foundation for future theory and model development, and may further be relevant in the context of action/intention prediction for (social) robotics and effective human–robot interaction.

## Introduction

When humans collaborate, they may work on their own tasks, share tasks, or communicate in various ways. Understanding such collaboration requires a comprehensive exploration from different perspectives, including, e.g., the control of sequential action (Norman and Shallice, 1986; Cooper and Shallice, 2000; Botvinick and Plaut, 2004) and interpersonal interaction (Argyle and Dean, 1965; Patterson, 1982; Sebanz, Bekkering, Knoblich, 2006; Marsh, Richardson, Baron, Schmidt, 2006; Paxton and Dale, 2013; Hessels, 2020; Hadley, Naylor, Hamilton, 2022). In this work, we are concerned with the interplay of vision for sequential action and vision for interpersonal coordination. The eye-movement system plays a crucial and multifaceted role in the context of human interaction, as gaze may be used to both gather information from the world or may be a relevant signal to others. For example, where one looks may serve to guide one’s actions (Land, Mennie, Rusted, 1999), it may help convey a message about maintaining one’s turn in conversation (Kendon, 1967), or one’s attention may be automatically attracted to novel events in the environment (Theeuwes, 1991). In this study, we explore gaze-action coupling, gaze-speech and gaze-gesture coupling, as well as the attraction of gaze by the other person’s action in the context of the same collaborative interaction.

A major theoretical framework on gaze-action coupling is that of visual routines theory (Ullman, 1996; Hayhoe, 2017), which emphasizes the fundamental role of vision in guiding human behavior by gathering knowledge about the world, allowing rewarding tasks to be executed (Hayhoe and Ballard, 2005, 2014). Gaze behavior is posited to be tightly linked to the evolving task execution, enabling individuals to extract task-specific information from different gaze fixations. Empirical support for this theory comes from Land, Mennie, Rusted (1999), Hayhoe (2000), and Pelz and Canosa (2001), who used wearable eye-tracking technology to show that gaze fixations are tightly linked to the current behavioral goal while few gaze fixations on task-irrelevant locations occurred. For example, while making tea, gaze tends to be allocated to the kettle, before one lifts the kettle (Land, Mennie, Rusted, 1999). More generally, gaze tends to precede the hand during manual aiming, with a consistent temporal coupling (see e.g., Helsen, Elliott, Starkes, Ricker ; 1998, Helsen, Elliott, Starkes, Ricker ; 2000). The task-related nature of gaze behavior has further been shown for, e.g., collision avoidance (Jovancevic, Sullivan, Hayhoe, 2006; Rothkopf, Ballard, Hayhoe, 2007; Jovancevic-Misic and Hayhoe, 2009; Tong, Zohar, Hayhoe, 2017), crowd navigation (Hessels, van Doorn, Benjamins, Holleman, Hooge, 2020c), foot control in rough terrain (Matthis, Yates, Hayhoe, 2018), and stair climbing (Ghiani, Van Hout, Driessen, Brenner, 2023).

While visual routines theory has significantly contributed to our understanding of the relationship between vision and sequential action, it has certain limitations when applied to the context of collaborative tasks. For example, it does not fully account for the attraction of gaze by external stimuli, such as the actions and communication attempts of other individuals. Indeed, this is what Hayhoe and Ballard (2014) make explicit in their review: “The focus [...] has been on endogenous attentional control of tasks, but a complete story has, in addition, to account for exogenous stimuli that can change the agent’s agenda” (p. R628). While visual routines theory describes how relevant visual information for current behavioral goals may be acquired from the world in sequential gaze fixations, it does not address interruptions in the execution of current tasks or the addition of tasks to the agent’s agenda by other individuals. In collaborative or competitive settings, the behavior of one individual can influence the subsequent actions and gaze allocation of another, leading to coordinated and mutually adjusted behavior (see e.g., Gergle, Kraut, Fussell ; 2004, Marsh, Richardson, Schmidt ; 2009, Gergle, Kraut, Fussell ; 2013, Coco, Dale, Keller ; 2018, Niehorster, Cornelissen, Holmqvist, Hooge ; 2019). Specifically, individuals may monitor the actions of the other person and show similar gaze patterns as for the individual performing the actions (Flanagan and Johansson, 2003).

Gaze behavior may not only serve as an information-gathering mechanism for an agent to complete its current task successfully but may also play an important role in communication during social interaction (see, e.g., Kendon ; 1967, Argyle and Cook ; 1976, Risko, Richardson, Kingstone ; 2016). During interaction, gaze behavior may fulfill both the role of gathering information (what is the other person conveying?) and as a communicative signal (see, e.g., Kingstone ; 2009, Risko, Laidlaw, Freeth, Foulsham, Kingstone ; 2012, Holleman, Hooge, Kemner, Hessels ; 2020). One’s gaze direction may, for example, play a crucial role in regulating conversational dynamics, in particular turn-taking behavior (Ho, Foulsham, Kingstone, 2015; Hessels, Holleman, Kingstone, Hooge, Kemner, 2019; Maran, Furtner, Liegl, Ravet-Brown, Haraped, Sachse, 2021; Wohltjen and Wheatley, 2021), as well as the expression of intimacy or asserting social control (Argyle and Dean, 1965; Kendon, 1967; Kleinke, 1986), and disambiguating expressions or following instructions (Hanna and Brennan, 2007; Macdonald and Tatler, 2013). Thus, gaze behavior plays an important role in the communicative context in relation to other modalities such as speech and gesture (see also Wagner, Malisz, Kopp ; 2014, Kendrick, Holler, Levinson ; 2023).

Ideally, one would want a model that integrates gaze-action coupling (as in Hayhoe and Ballard ; 2005, Hayhoe and Ballard ; 2014), gaze for communication, and exogenous attraction of gaze. Such a model can be achieved at various levels of abstraction and for various purposes, including understanding vision for action or implementation in social robotics (compare, for example, Huang and Mutlu ; 2012, Hayhoe and Ballard ; 2014). We believe it is methodologically premature to deliver, for example, an extended model of Hayhoe and Ballard (2014) to account for exogenous attraction of gaze, let alone gaze for communication in the context of human interaction. Although wearable eye trackers allow studying gaze behavior in the context of daily behavior and have been applied to the study of face-to-face interaction (e.g., Andrist, Collier, Gleicher, Mutlu, Shaffer ; 2015, Rogers, Speelman, Guidetti, Longmuir ; 2018, Haensel, Smith, Senju ; 2022), analyses have often been limited in scope or have mainly depended on manual annotation, which limits their widespread application (see also Hessels, Niehorster, Holleman, Benjamins, Hooge ; 2020b, Valtakari, Hooge, Viktorsson, Nyström, Falck-Ytter, Hessels ; 2021). Thus, the necessary empirical foundation to develop such integrated models may be missing at present. This empirical foundation can be provided by investigating gaze allocation across various collaborative interactions, which may vary from being very task-oriented to more unconstrained conversation.

In this paper, we present an extended analysis of a dataset by Hessels, Teunisse, Niehorster, Nyström, Benjamins, Senju, Hooge (2023), who investigated gaze behavior during face-to-face collaboration using a Lego Duplo model-copying task based on Ballard, Hayhoe, Pelz (1995). This collaboration is interesting because it uses a copying task that is well studied for individuals and elicits clear task-oriented gaze behavior (Ballard, Hayhoe, Pelz, 1995). The copying task was completed in collaboration, thus allowing for action–observation-related gaze patterns or exogenous attraction of gaze to emerge. Moreover, non-verbal communication was emphasized by restricting verbal communication in some of the conditions, thus potentially eliciting gestures and gesture-related gaze patterns. Finally, the study by Hessels, Teunisse, Niehorster, Nyström, Benjamins, Senju, Hooge (2023) combines state-of-the-art wearable eye trackers with fully automated analysis of gaze behavior, with video and audio recordings that could be annotated for speech, gesture, and task-related behavior.

Hessels, Teunisse, Niehorster, Nyström, Benjamins, Senju, Hooge (2023) investigated (the coordination of) gaze behavior during the Duplo model-copying task, varying whether all blocks in a model were visible to both participants (“visible” models) and when some blocks were hidden from one participant’s view (“hidden” models), which would prompt more communication. Moreover, it was varied whether participants were allowed to talk or not. Participants primarily focused on the model and build area, rarely looking at their collaborator’s face, consistent with prior research on dyadic collaboration. Interestingly, while individual measures of gaze behavior did not seem to vary significantly with communication mode, pair-based gaze coordination did. Specifically, when verbal communication was allowed, participants coordinated their gaze more closely for the hidden model condition, which suggested improved coordination between participants. However, this enhanced coordination did not translate into faster model completion or fewer mistakes. The study concludes that task execution and corresponding gaze behavior and coordination are influenced by the availability of visual information and the mode of communication, though the latter did not necessarily improve task outcomes.

In the present study, we shift our focus to the temporal relation between gaze, manual actions, gestures, and speech. Specifically, we use the dataset by Hessels, Teunisse, Niehorster, Nyström, Benjamins, Senju, Hooge (2023) to investigate patterns of (1) gaze-action coupling, i.e., visually guided execution of task steps, (2) gaze-gesture and gaze-speech coupling, or what might be considered gaze for communication, and (3) coupling between one person’s gaze and another person’s manual actions, gestures, or speech, or exogenous attraction of gaze. Importantly, our goal is to describe and quantify these patterns as they occur in the context of face-to-face dyadic collaboration. Finally, we investigate how well gaze behavior can be inferred from one’s own manual actions, gestures, or speech behavior, as well as the behavior – gaze, actions, gestures, speech – of the collaborator. This analysis reveals the relative contribution of gaze-action coupling, gaze-gesture and gaze-speech coupling, and exogenous attraction of gaze to the overall gaze behavior in dyadic collaborative interaction.

**Fig. 1:**
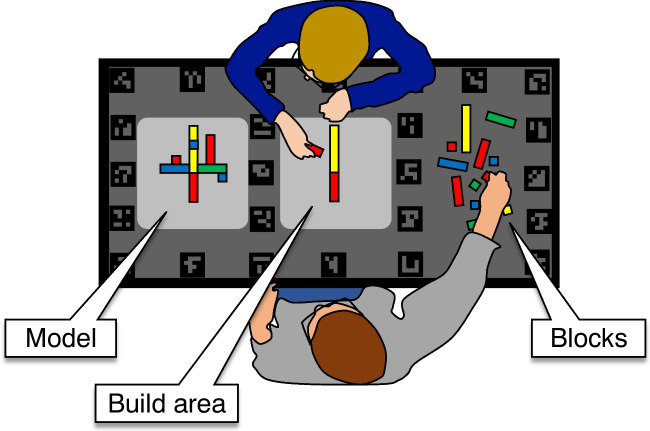
Schematic top view of the setup. Dyads copied a model in the build area, using the blocks provided in a separate area. Figure adapted from Hessels, Teunisse, Niehorster, Nyström, Benjamins, Senju, Hooge (2023)

By examining the interaction, communication, and gaze allocation during task execution, we aim to enhance our understanding of the dynamics of collaborative behavior. The findings of this study have implications for understanding the limitations of current models of gaze behavior in the context of interaction, as well as for developing more comprehensive models of collaborative interactions as a whole. The application for modeling interactive behavior is in line with a general trend of considering perception and cognition from an interactive perspective as opposed to an individual perspective (e.g., Pickering and Garrod ; 2004, Hutchins ; 2010, Fusaroli and Tylén ; 2016, Dingemanse, Liesenfeld, Rasenberg, Albert, Ameka, Birhane,... Wiltschko ; 2023). More practically, our findings are relevant for (social) robotics. Some examples are (1) the development of models of human-like gaze behavior for virtual avatars and social robots that incorporate perception, multimodal communication and interactive behavior (cf. Huang and Mutlu ; 2012, Ruhland, Peters, Andrist, Badler, Badler, Gleicher,... McDonnell ; 2015, Mihoub, Bailly, Wolf, Elisei ; 2016, Admoni and Scassellati ; 2017), (2) action or intention prediction in human–object or human–human interaction (e.g., Ragusa, Furnari, Livatino, Farinella ; 2020, Ragusa, Furnari, Farinella ; 2022), and (3) development of task structures for effective human–robot interaction in, e.g., industrial settings (Villani, Pini, Leali, Secchi, 2018; Zhao, Henrichs, Mutlu, 2020).

## Methods

The present study extends Hessels, Teunisse, Niehorster, Nyström, Benjamins, Senju, Hooge (2023) with novel analyses. We therefore summarize essential information about (1) participants, (2) setup, apparatus, and task, and (3) the gaze and speech data from the original study. For additional details, we refer readers to Hessels, Teunisse, Niehorster, Nyström, Benjamins, Senju, Hooge (2023). After this summary, the annotations and analyses novel to the present study are described in detail.

### Participants

Recordings were available from 19 dyads arranged in nine female–male, four female–female, five male–male, and one male-unspecified dyads. Mean age was 23.3 years (SD = 3.0 years, range 19–31 years). The mean age difference within the dyads was 2.8 years (SD = 1.9 years, range 0–7 years). Participants confirmed that they did not know each other well and spoke the same language (English or Dutch). All participants gave written informed consent and the study was approved by the Ethics Committee of the Faculty of Social and Behavioural Sciences of Utrecht University (protocol number 22-0206).

### Setup, apparatus, and task

Participants were seated at either end of a table (see Fig. 1) and were instructed to copy a Lego Duplo model together. The table was divided into an area for the Duplo model to be copied by the dyad (*model*), an area that contained the Duplo building blocks (*blocks*), and an area where the Duplo model was to be built (*build area*). Each participant was fitted with a Pupil Invisible eye tracker (Pupil Labs, Tonsen, Baumann, Dierkes ; 2020) to record gaze direction at an effective frequency (Hooge, Niehorster, Hessels, Benjamins, Nyström, 2022) across participants of 219.23 Hz (range 217.26–225.21 Hz), and 30.83 Hz for the scene cameras (range 30.72–30.94 Hz). A Logitech BRIO 4K Stream Edition webcam (version 1.0.40) recorded a full HD top view of the table at 30 Hz.

The experiment consisted of eight trials, during which dyads had to simultaneously copy a Duplo model consisting of 26 individual Duplo blocks (varying in color and size). Participants were instructed to copy the model as quickly and accurately as possible. There were no instructions or restrictions on how the model ought to be built, e.g., with respect to turn-taking or strategy. The only restrictions were that participants could not move or disassemble the model, or get up from their chairs. Two different model types were used: (1) two-tiered models in which all blocks were visible from each participant’s perspective (‘visible’ models), and (2) three-tiered models in which some blocks were hidden from each participant’s view (‘hidden’ models). Depictions of these model types are given in Hessels, Teunisse, Niehorster, Nyström, Benjamins, Senju, Hooge (2023) and schematics for all models can be found at https://osf.io/2mc8p/. For half of the trials, participants were not allowed to communicate verbally with each other (‘no talking’). This yielded four possible trial types: (1) visible model, no talking, (2) visible model, talking, (3) hidden model, no talking, (4) hidden model, talking. Each trial type was conducted twice. Each block of four trials contained all possible trial types and the exact order was pseudorandom across dyads. Example videos for each trial type can be found online at https://osf.io/2q6f4/. Overall, hidden models took roughly twice as long to complete than visible models (approximately 160 s vs. 80 s), while performance did not depend on whether talking was allowed or not. Very few mistakes were made overall (for details, see Fig. 4 in Hessels, Teunisse, Niehorster, Nyström, Benjamins, Senju, Hooge ; 2023). The experiment took approximately 30 min in total.

### Signal processing

#### Gaze behavior

Fiducial markers (Garrido-Jurado, Muñoz-Salinas, Madrid-Cuevas, Marín-Jiménez, 2014) were placed on the table to allow mapping of the gaze position from the eye tracker scene camera’s coordinate system (head-fixed) to the coordinate system of the table (world-fixed). This procedure transforms a gaze position in pixels in the eye tracker scene camera to a position in the 2D plane of the table in millimeters, when gaze is directed towards the table. This was done using code from the GlassesValidator tool for wearable eye trackers (Niehorster, Hessels, Benjamins, Nyström, Hooge, 2023). An example video of the gaze data after mapping to the table coordinate system can be found online at https://osf.io/2q6f4/. In addition, face detection software (CVZone, git revision a6d0d6d, building on MediaPipe) was run on the scene camera videos of both eye trackers. All signals were synchronized to the top camera and resampled to 30 Hz. Synchronization was reliable to approximately one frame of the top view camera (i.e., 33 ms).

When gaze was on the table, it was assigned to one of three possible areas of interest (AOIs): the block selection area, the build area, and the model. When gaze was not on the table, it was mapped to the face AOI when the gaze position did not exceed 150 pixels from the center of the bounding box around a face in the eye tracker scene camera image. When gaze was not on one of the table AOIs or on the face, it was assigned to the ‘none’ AOI. Thus, a participant’s gaze could be on one of five AOIs: model, build area, blocks, the other person’s face, or none (see Fig. 3 in Hessels, Teunisse, Niehorster, Nyström, Benjamins, Senju, Hooge (2023) for more details about the gaze-data processing pipeline). Precision and accuracy were deemed good enough to allow the mapping of gaze to these AOIs. Exact numbers are reported in Hessels, Teunisse, Niehorster, Nyström, Benjamins, Senju, Hooge (2023) according to recent reporting standards (Dunn, Alexander, Amiebenomo, Arblaster, Atan, Erichsen,... Sprenger, 2023).

#### Speech behavior

Speech was annotated for the first two trials during which speech occurred, i.e., a visible model and talking trial, and a hidden model and talking trial. The motivation to annotate only the first minute was two-fold. First, we wanted an equal amount of annotated data for each trial, regardless of how long the dyad took to complete copying the model. The shortest time to complete a model was approximately 1  min. Second, we chose to annotate the first minute, and not, e.g., the last minute, as the behavior in the last part of the trial could vary substantially. For example, in some cases, mistakes had to be repaired, or there were discussions about whether the model was completed properly. In the first minute of each trial, generally similar collaborative behavior was shown across dyads.

One of the scene camera audio streams was annotated in ELAN 6.4 (The Language Archive, 2022) for when each participant spoke. Each utterance was defined by a start and end time. The speech was annotated independently by authors RH and MT, using only the audio stream such that the annotator could not see which model was being copied. In what follows, the annotations of author RH are used, but the pattern of results and conclusions are identical when using the annotations of author MT (i.e., the reported outcomes are not dependent on whose annotations we use).

#### Manual actions and gestures

The first minute of the first four trials in the experiment was annotated for manual actions and gestures, again such that we had an equal amount of annotated data for each trial, regardless of how long the dyad took to complete copying the model. This included one trial for every possible unique combination of talking/no talking and model type. Manual actions and gestures were annotated by author SB from the top camera recordings using ELAN 6.4 (The Language Archive, 2022). Iterative discussions and revisions to the coding scheme were conducted between the authors. The following manual actions were annotated:

1. Grab. The action starts the moment the hand is inside the blocks area on the table and ends when no hands are in the block area, having grabbed at least one block.
2. Place. The action starts when the participant initiates the movement to place the block into the build area and ends when the block is attached to the plate or another block in the build area and the hand lets go of the block. If multiple blocks are placed, the first placement ends when the movement to place the second block starts.
3. Remove. The action starts the moment the participant grabs the block from the build area and ends when the block is removed from the plate or another block in the build area.
4. Drop. The action starts when the participant moves the hand to place a block aside or back at the block area and ends when the hand lets go of the block.

The following gestures were annotated:

1. Point. Any pointing gesture performed to attract attention to a specific location or specific block(s).
2. Ask, verify, or check. Any gesture, except for pointing, performed to ask about, verify, or check. Examples are a thumbs up to verify correct placement, or a gesture signaling uncertainty about a placement.
3. Instruct. Any gesture, except for pointing, performed to indicate that a correction or an action is needed. Examples are gestures indicating orientation of a block placement or the size of a block to use.

Gestures were annotated from the moment the movement was initiated until the gesture disappeared.

**Fig. 2:**
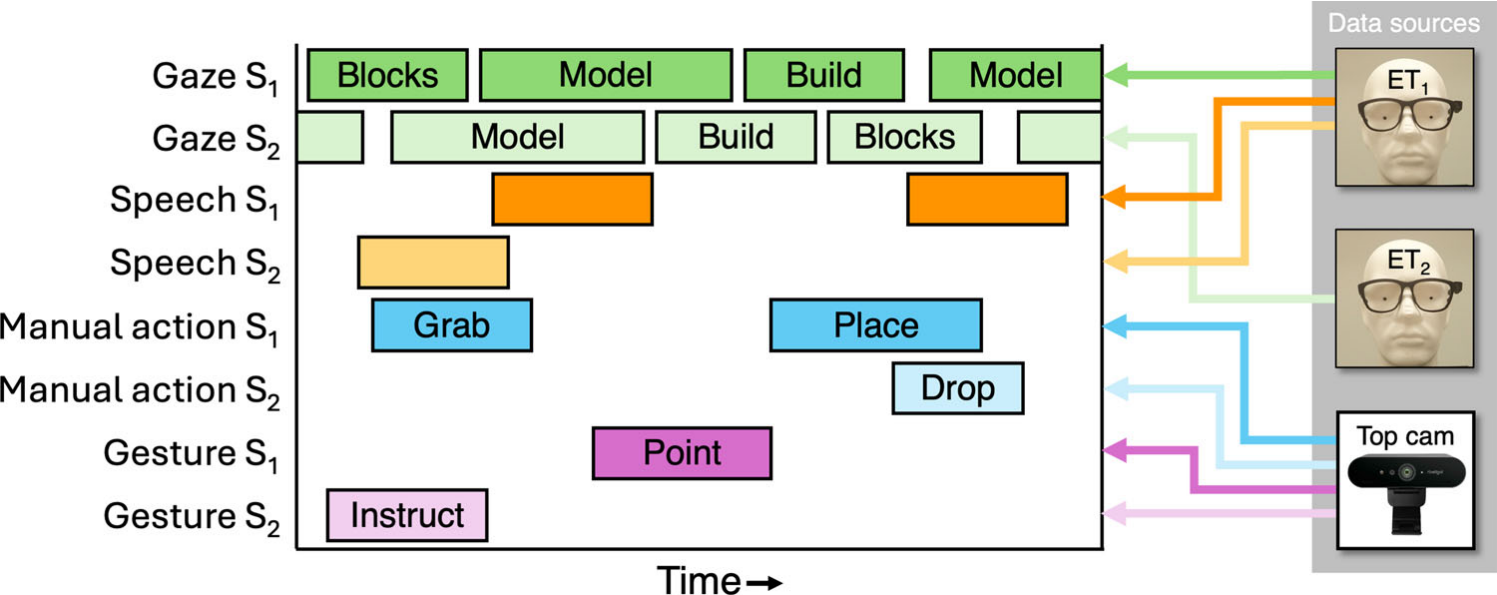
Schematic overview of the data structure and its sources. S1 and S2 refer to subjects 1 and 2 in each dyad, respectively. The gaze (*green bars*) was categorized as being on the blocks AOI, model AOI, build AOI, or face AOI, using the eye-tracker data and scene videos and then expressed in the top camera time. The presence of speech (*orange bars*) was annotated from the audio stream of one of the eye tracker scene videos, and timestamps were converted to top camera time. Manual actions (*blue bars*) were categorized as grab, place, drop, or remove actions from the top camera. Gestures (*purple bars*) were categorized as point, ask, or instruct gestures from the top camera. For every time point at which no AOI is fixated, or no action, gesture, or speech episode took place, ‘none’ was recorded in the datafile

#### Combining signals

Speech annotations consisted of onsets and offsets determined from the 44.1-kHz audio file. Annotations for manual actions and gestures consisted of onsets and offsets determined from the 30-Hz top-view camera. Gaze data consisted of 30-Hz signals with the timestamp transformed to the top view camera time. They were then combined with the annotated manual actions, gestures, and speech data. This yielded a data set containing categorical gaze data (model, build area, blocks, face, and none AOI), manual actions (grab, place, remove, drop), gestures (point, ask, instruct) for 4 x 1 min from 19 dyads (i.e., 38 participants). In addition, for half (two out of four) of the trials, when verbal communication was allowed, the data set contained categorical speech data (talking, quiet). Specifically, the data were combined as follows.

First, a new 60-Hz signal was constructed for the period that categorical gaze data, speech annotations (if applicable), and annotations for manual actions and gestures were available. Second, for each sample in this new time signal, the gaze data (i.e., AOI label) closest in time was assigned. Given that the gaze data was originally at 30 Hz, this yielded a maximum offset of 16.7 ms. Third, for each gaze sample in this new time signal, we determined whether it fell within an episode of a manual action, gesture, and utterance (speech). If so, their respective categories were assigned to this time point. If not, manual actions, gestures, or speech were assigned the ‘none’ label. Combined data files are available at https://osf.io/2q6f4/. Figure 2 depicts a schematic overview of the data structure, including the sources of the data.

### Data analysis

In the present study, we aim to uncover patterns of (1) gaze-action coupling, (2) gaze-gesture and gaze-speech coupling, and (3) coupling between one person’s gaze and another person’s manual actions, gestures, or speech. We investigate these couplings with two different types of gaze measures:

1. Relative total dwell time on an AOI while a manual action, gesture, or speech episode occurs. This is computed on a per-trial basis and a per-participant value is determined by averaging across the four trials for which we have data.
2. Proportion of looks to the various AOIs as a function of time until the onset of a manual action or gesture. The onset of an action or gesture is defined as the time of the first sample during which the action or gesture occurs. We report the proportion of looks to each AOI from -3 to +3 s relative to the onset of each gesture or action across all instances of a particular action or gesture type, regardless of which participant made the action or gesture. The choice for the -3 to +3-s window was determined empirically. For all analyses reported below, the gaze behavior at -3 or +3 s from action or gesture onset was around the trial average.

The goal of our analyses is two-fold. First, we want to describe the patterns of gaze-action, gaze-gesture, gaze-speech coupling, and coupling between one person’s gaze and another person’s manual actions, gestures, or speech in our dataset and assess their relative magnitude. We achieve this with descriptive statistics and visualization techniques, following advice from, e.g., Rousselet, Pernet, Wilcox (2017). Second, after highlighting the patterns we find, we want to determine how tightly gaze is coupled to one’s own manual actions, gestures and speech behavior, or those of the interacting partner in the collaborative model-copying task. To quantify this, we take a modeling approach to infer gaze behavior from the manual actions, gestures, and speech behavior as well as the behavior of the interacting partner. We constructed six models to infer the gaze location during the entire 1-min interaction in four trials, based on either (1) one’s own manual actions, (2) one’s own gestures, (3) one’s own speech episodes, (4) the other person’s manual actions, (5) the other person’s gestures, or (6) the other person’s speech episodes that occurred. Data from 18 pairs were used to infer the gaze behavior of the 19th pair, using a leave-one-out cross-validation procedure. The performance of each model was expressed as the proportion of correctly inferred gaze locations (i.e., correct AOI inferred across all samples of the gaze data) across the four 60-s trials.

**Fig. 3:**
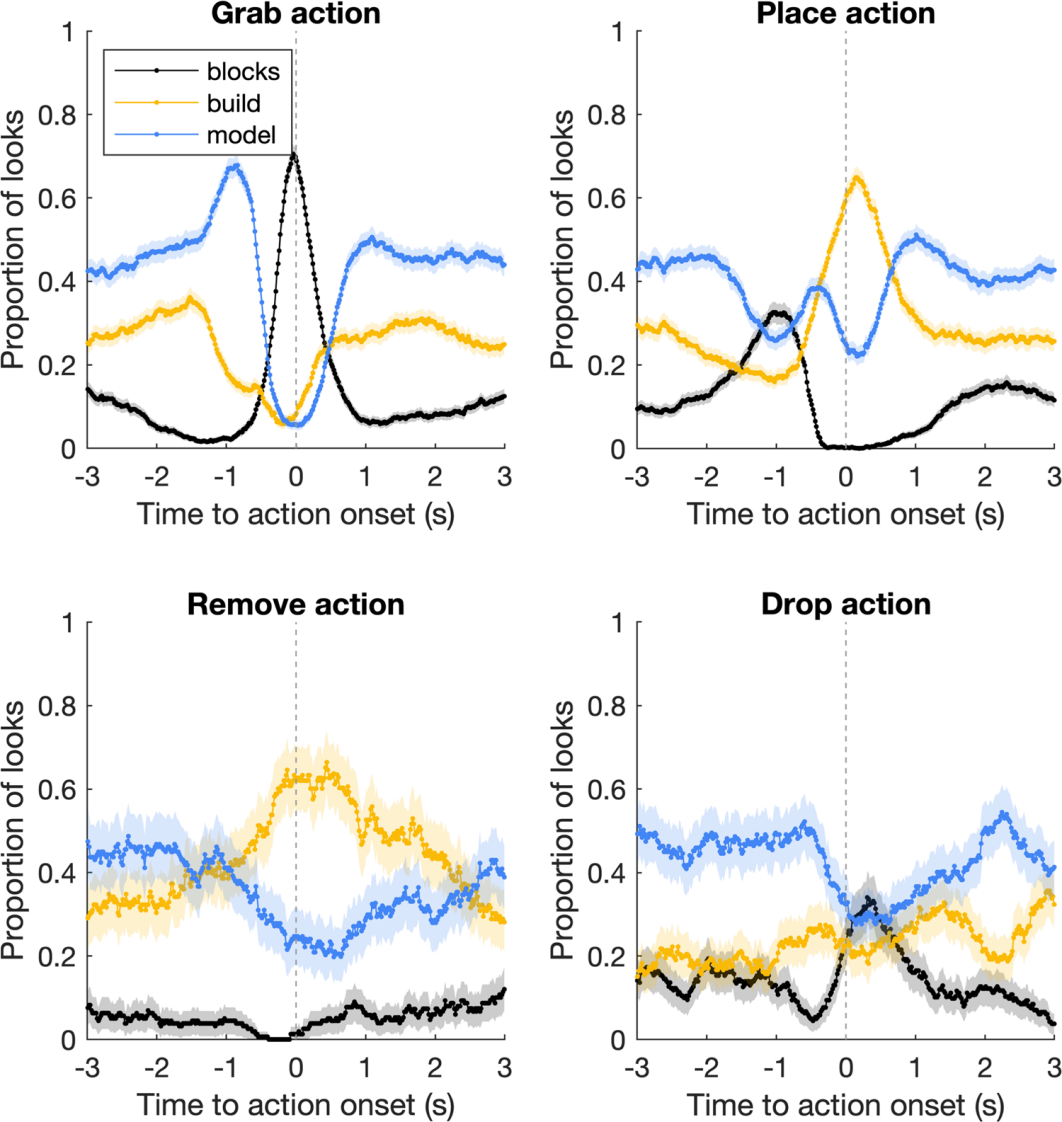
Coupling between a person’s own gaze behavior and manual actions. The panels depict the proportion of looks to each Area of Interest (AOI; blocks, build, and model area) from -3 to 3 s relative to the onset of the grab (top left), place (top right), remove (bottom left), and drop (bottom right) actions. The lines depict the proportion of looks to each AOI across all instances of a particular action (i.e., all actions of all participants). The shaded areas represent 95%CI of the mean across all action instances

Specifically, we constructed six models based on the conditional probability of fixating an AOI in -3 to +3 s around the onset of (1) one’s own manual actions, (2) one’s own gestures, (3) one’s own speech episodes, or the onset of (4) the other person’s manual actions, (5) the other person’s gestures, or (6) the other person’s speech episodes. The process of inferring gaze behavior for each of these six models was as follows:

1. Leave out the data of one of the 19 pairs.
2. For the other 18 pairs, determine the conditional probability of fixating each of five AOIs as a function of time ([-3, 3] s, at 16.7-ms intervals, or 60 Hz) to the onset of one’s (1) own action, (2) gesture, or (3) speech episode, or the onset of the (4) other person’s action, (5) other person’s gesture or (6) other person’s speech episode. As an example, consider the case of the ‘own action model’. A conditional probability function similar to those depicted in Fig. 3 is constructed, except it is now based on 18 pairs instead of all 19 pairs. Around the grab action onset (top left panel), the probability of fixating the blocks AOI is highest. Thus, for the 19th pair, the model might predict the blocks AOI to be the likely AOI to be fixated around the grab action onset.
3. For the 19th pair, iterate across all gaze samples (i.e., each recorded gaze location at 60 Hz for four 60-s trials). For each gaze sample, determine whether one or more onsets (actions, gestures, speech episodes) occur within 3 s (earlier or later) relative to that gaze sample. Across all onsets within that window, average the conditional probabilities of fixating each of the five AOIs at this time point relative to the onsets. For example, if at time point *t* a grab action has occurred 0.5 s ago and a place action is upcoming in 1 s, find the likelihood of fixating each AOI relative to those actions at their temporal offsets (-0.5 s and 1 s) and average them. Then find the maximum probability and assign the corresponding AOI label to the current gaze sample.
4. For all gaze samples with no onsets of actions, gestures, or speech within 3 s relative to that gaze sample, assign the most likely fixated AOI (model AOI) across all 18 pairs.
5. For this 19th pair, determine the percentage of correctly labeled gaze samples for each of the four 60-s trials, by comparing the model inference to the actual fixated AOI by the two participants in that pair.
6. Re-run the procedure leaving out the data of another one of the 19 pairs, until all pairs have been left out once.
7. Average the model performance across the four trials per pair, and across all pairs.

To interpret the performance of a particular model (e.g., the ‘own action model’) in inferring gaze behavior, we compare it against a benchmark model. Our benchmark model used the most likely gaze location (i.e., majority class) across the five AOIs (model, build area, blocks, face, and no AOI) for 18 pairs to derive a predicted gaze location for the 19th pair. In other words, this model assumes that everyone always looks at the model AOI, as it was looked at the most across every possible combination of 18 pairs. This means that the performance of the benchmark model for the 19th pair is equal to that pair’s proportion of time spent looking at the model AOI. In comparing each of the six models described above with the benchmark, we can learn how much of the gaze behavior can be additionally predicted based on one’s own manual actions, gestures, or speech episodes, or by the actions, gestures, or speech episodes of the person one interacts with.

## Results

### Descriptive statistics of gaze, manual actions, gestures, and speech

Before we describe patterns of within-person gaze-action, gaze-gesture, and gaze-speech coupling, we first report descriptive statistics of each of these behaviors across all individuals. Table 1 summarizes the gaze behavior, manual actions, gestures, and speech by the frequency that each behavior occurred and the proportion of time that behavior took up within a 60-s trial. Regarding the gaze data, participants spent most of their time looking at the table AOIs, and very little at the face of the other person. Regarding manual actions, approximately 8–9 grab and place actions occurred each minute, taking more than half of the annotated time (approximately 13 s for grab and 20 s for place actions out of the total 60 s). Remove and drop actions occurred infrequently. Regarding gestures, point gestures were the most common, while gestures meant to ask, verify, check, or instruct were infrequent (< 1 per trial). For the trials in which verbal communication was allowed, participants spoke for approximately one-fourth of the annotated 60 s making an average of 12 utterances. In addition, Table 1 depicts the total number of occurrences of manual actions and gestures, which are relevant to the analyses of the proportion of looks to an AOI as a function of time to action or gesture onset presented below.

**Table 1:** Descriptives statistics for gaze behavior, manual actions, gestures, and speech

	Variable	Proportion of time	Frequency	Occurrences
Gaze	Blocks	0.11 (0.07–0.14)	14.50 (7.50–22.75)	
	Build area	0.25 (0.17–0.33)	37.38 (20.50–60.00)	
	Model	0.47 (0.32–0.56)	35.12 (23.50–58.25)	
	Face	0.00 (0.00–0.02)	1.12 (0.00–5.00)	
	None	0.17 (0.08–0.38)	n.a.	
Actions	Grab	0.21 (0.12–0.41)	9.25 (5.50–13.75)	1393
	Place	0.33 (0.18–0.46)	8.50 (5.25–14.00)	1346
	Remove	0.01 (0.00–0.04)	1.00 (0.00–3.00)	158
	Drop	0.01 (0.00–0.06)	1.12 (0.00–7.25)	222
	None	0.40 (0.18–0.66)	n.a.	
Gestures	Point	0.04 (0.00–0.20)	1.62 (0.00–6.50)	299
	Ask, verify, check	0.00 (0.00–0.02)	0.25 (0.00–1.25)	41
	Instruct	0.00 (0.00–0.04)	0.25 (0.00–2.50)	52
	None	0.95 (0.79–1.00)	n.a.	
Speech	Talking	0.24 (0.05–0.62)	12.00 (3.00–24.50)	
	Quiet	0.76 (0.38–0.95)	n.a.	

Medians and range across participants for the proportion of time and frequency (number of instances per minute) were first averaged on a per-participant basis: four trials for gaze, manual actions, and gestures, and two trials for speech. The total number of occurrences are reported for gestures and manual actions

### Within-person gaze-manual action coupling

In our Duplo model-copying experiment, four task-related manual actions were defined: grab, place, remove, and drop actions. For each of these actions, the relevant AOIs are the blocks, build area, and model on the table. Figure 3 depicts the proportion of looks to these three AOIs as a function of time to the onset of a manual action for all individuals. Based on Ballard, Hayhoe, Pelz (1995), we expect participants to look at the relevant areas just before a manual action is carried out there.

We consider first the grab action (top left panel). Just before the onset of the grab action, there is a peak in the proportion of looks to the model AOI (blue line), followed by a peak for the blocks AOI (black line) at around 0 s to action onset. From 0 s, gaze to the blocks AOI drops, while gaze to the build (yellow line) and model (blue line) areas increases. These patterns seem to match two of the common strategies described in Ballard, Hayhoe, Pelz (1995), namely what they call the model-pickup-model-drop and model-pickup-drop strategies (cf. their Fig. 4). Note that ‘pickup’ and ‘drop’ in Ballard, Hayhoe, Pelz (1995) do not refer to actions, but to a concurrent gaze location and manual action at what we call the blocks and build AOIs, respectively. For the place action (top right panel), we see successive peaks for the blocks area (black line) around -1 s, the model area just before 0 s (blue line), the build area around 0 s (yellow line) and the model area around 1 s (blue line). This matches well the pickup-model-drop strategy described in Ballard, Hayhoe, Pelz (1995). For both the grab and place actions, the changes in the proportion of looks to the various AOIs as a function of time are substantial, ranging from 50-60% (model AOI for grab action and build AOI for place action) and even exceeding 70% in the case of the blocks AOI for the grab action.

**Fig. 4:**
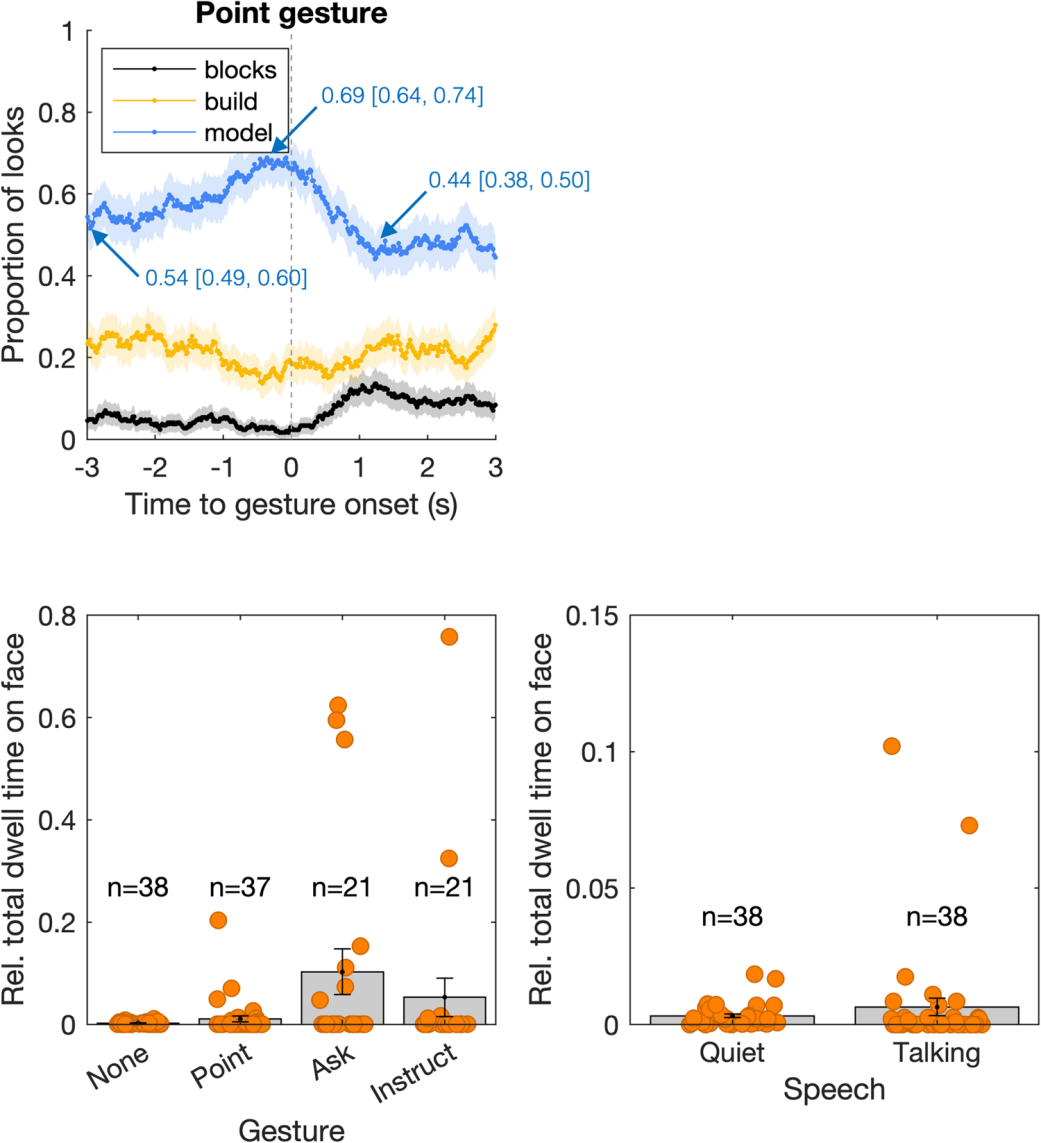
Coupling between a person’s own gaze behavior and gestures and speech. The *top left panel* depicts the proportion of looks to each area of interest (AOI; blocks, build, and model area) from -3 to 3 s after the onset of the point gesture. The *lines* depict the proportion of looks to each AOI across all instances of the point gesture, with the *shaded areas* representing 95%CI of the mean across all gesture instances. The values depicted in the *top left panel* represent the proportion of looks to the model AOI at the start of the 3-s window prior to gesture onset, as well as the maximum and minimum values with corresponding 95% confidence intervals. The *bottom left panel* depicts the relative total dwell time on the face AOI when a point gesture, ask, verify, or check gesture (labeled ‘ask’), instruct gesture, or no gesture took place. The *bottom right panel* depicts the relative total dwell time on the face AOI during episodes where participants were quiet or spoke. *Grey bars* represent the means across all participants with standard errors of the mean. *Orange markers* represent averages for each participant. The *numbers* indicate how many participants contribute to each average. Note that the limits of the vertical axis differ between the bottom two panels

For the remove action (bottom left panel), we see a clear decrease in the proportion of looks at the model just before and after action onset (blue line) and a concurrent increase for the build area (yellow line). For the drop action (bottom right panel), we observe a decrease for the model area (blue line) around 0 s and a concurrent increase for the blocks area (black line). The peak for the blocks area is less pronounced than for the grab action, and seems to occur slightly later. It should be noted that the drop action, unlike the grab action, could also be performed without visual guidance, because the exact location of the drop is inconsequential. For the remove and drop action, the changes in the proportion of looks to the various AOIs are somewhat smaller than for the grab and place actions, but still substantial, i.e., about 40% to the build AOI for the remove action or about 30% to the blocks AOI for the remove action.

In summary, we observe a strong relation between the manual action and the gaze location in the world around the action onset, which matches earlier work (e.g., Ballard, Hayhoe, Pelz ; 1995, Land, Mennie, Rusted ; 1999, Hayhoe ; 2000).

### Within-person gaze-gesture and gaze-speech coupling

Regarding gaze-gesture and gaze-speech coupling, we observed two relevant patterns. The top left panel in Fig. 4 depicts the proportion of looks to the three AOIs on the table as a function of time to the onset of a pointing gesture for all individuals. There is a peak in the proportion of looks to the model AOI just prior to the gesture onset (around -0.5 s). After gesture onset (0 to 1 s), gaze to the model AOI decreases, while gaze to both the build area and the blocks increases slightly. While the gaze-gesture pattern is evident, it seems less pronounced than the gaze-action coupling described above and depicted in Fig. 3. We therefore sought to quantify the magnitude in more detail. The values depicted in the top left panel in Fig. 4 indicate the proportion of looks to the model AOI at the start of the 3-s window prior to gesture onset, as well as the maximum and minimum values. The proportion of looks to the model AOI increases by about 15% around the time of the gesture onset compared to 3 s before the gesture. The proportion of looks to the model AOI then decreases after the gesture onset, to about 10% less than 3 s before the gesture onset. Overall, the magnitude of the gaze-gesture coupling is about 25% of the looks to the model AOI.

The bottom left panel in Fig. 4 depicts the relative total dwell time on the face AOI during the 1-min trial segments as a function of whether and which gesture was being made. That is, the time spent looking at the face AOI is expressed as a proportion of the time when a particular gesture is made, or when no gesture is made. We consider the total time on the face during the entire gesture, as the patterns for timing relative to gesture onset were not very informative. Overall throughout the experiment, and seen here when no gesture is made, the relative total dwell time to the face is very low (< 2%). However, this seems to increase during gestures, specifically for the ask type of gestures. Note, however, that this is only the case for some, but not all participants (as can be seen from the orange markers).

One might be inclined to conduct statistical tests on these data to lend credence to the difference in gaze to faces between the ask type of gesture and other or no gestures. However, this is not straightforward. Regular *t* tests are not appropriate, as the distributions of relative total dwell time to the face are highly non-normal. A non-parametric variant (e.g., the Wilcoxon signed-rank test) does not do justice to the orders of magnitude difference for some participants. Moreover, there are quite some missing values (17 out of 38) for those participants who did not make an ask type of gesture; Relative total dwell time during a gesture cannot be computed for these participants. One solution to this problem is to compute the shift function to compare two distributions, which can describe how two distributions differ rather than only whether their means are shifted according to some criteria of statistical significance (Rousselet, Pernet, Wilcox, 2017). The shift function describes the differences between two distributions for each decile (i.e., 10% of the participants). The error around these decile-differences can be quantified using bootstrapping.

We computed the shift function (using the *shifthd_pbci* MATLAB function from Rousselet, Pernet, Wilcox ; 2017) for the comparison of none vs. ask type of gesture, and for the point vs. ask type of gesture (i.e., comparing the ‘ask’ data in the bottom left panel in Fig. 4 with the ‘none’ and ‘point’ data). We find that a small subset of participants looks substantially longer at the face during an ask type of gesture than when making a point gesture or no gesture. Specifically, we find that the two distributions of relative total dwell time to the face differ in a robust fashion from the 7th to 9th decile, but not for the lower deciles. We find a higher relative total dwell time to the face AOI during an ask gesture than during no gesture for the 7th to 9th decile of 0.07 (95% CI [0.00, 0.33]), 0.22 (95% CI [0.02, 0.53]), and 0.49 (95% CI [0.10, 0.60]), respectively. Also, we find a higher relative total dwell time to the face AOI during an ask gesture than during a point gesture for the 7th to 9th decile of 0.08 (95% CI [0.00, 0.36]), 0.23 (95% CI [0.02, 0.51]), and 0.42 (95% CI [0.05, 0.56]), respectively.

The bottom right panel in Fig. 4 depicts the relative total dwell time on the face AOI as a function of whether participants were quiet or talked. It seems that there is no obvious relation between gaze to the face of the other person and one’s episodes of speech. Again, analyses for timing relative to utterance onset did not reveal noteworthy patterns.

### Exogenous attraction of gaze

Exogenous attraction of gaze was operationalized as the coupling between one’s gaze and the other person’s manual actions, gestures, or speech (i.e., between-person coupling). We consider four possible patterns. First, we consider the relation between one’s gaze behavior as a function of another person’s place or remove actions. We consider these actions, as they can be considered as relevant attention attractors that one might need to process to update what the possible next steps in the model-copying task might be. Second, we consider one’s gaze behavior in relation to another person’s gesture and their speech.

**Fig. 5:**
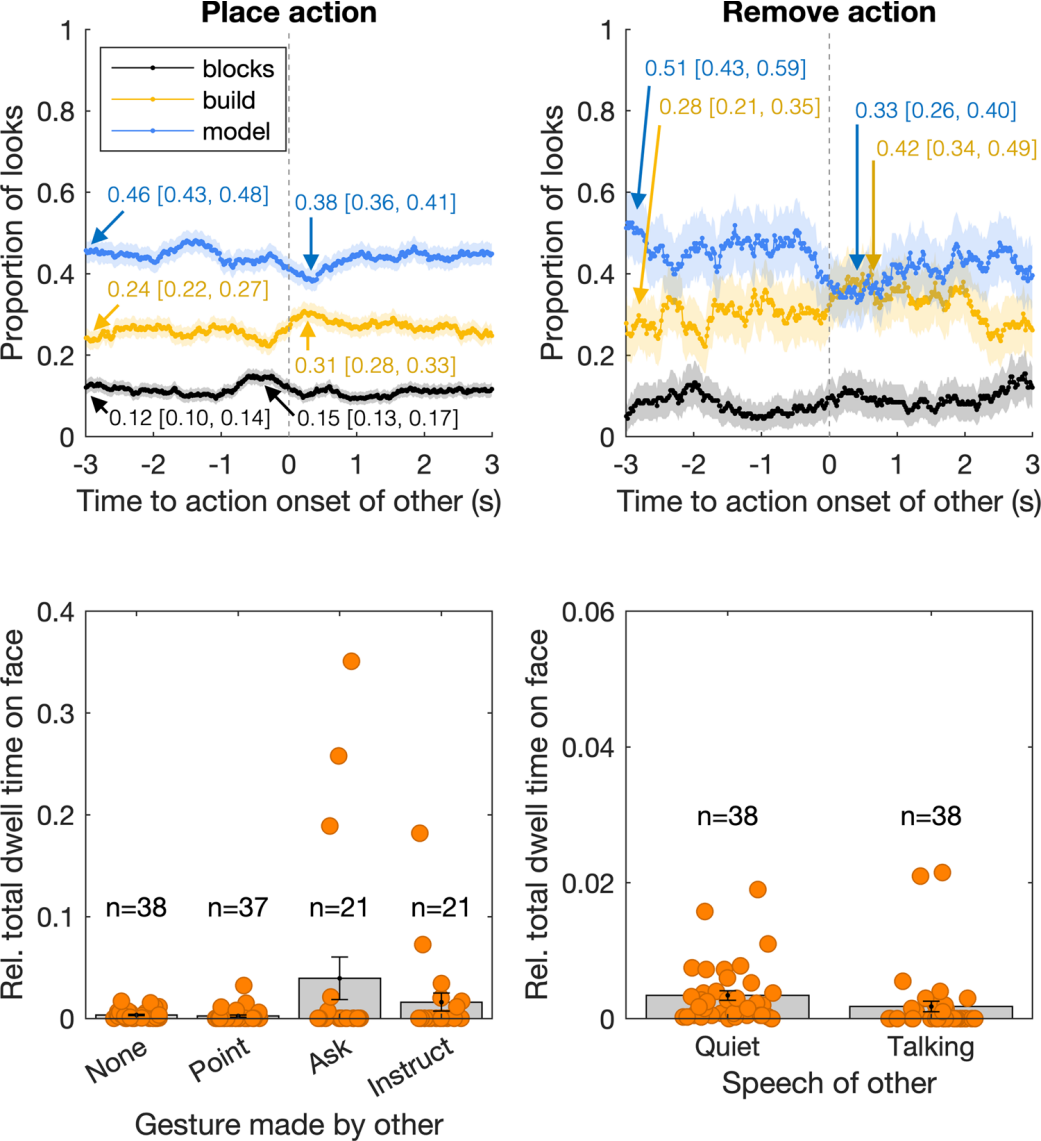
Between-person coupling between one person’s gaze behavior and the other person’s manual actions, gestures, and speech. The *top panels* depict the proportion of looks to the areas of interest (AOI) on the table (blocks, build, and model area) from -3 to 3 s relative to the onset of the interaction partner’s place (*top left*) and remove (*top right*) actions. The *lines* depict the proportion of looks to each AOI across all instances of the actions, with the *shaded areas* representing 95% CI of the mean. The values depicted in the top panels represent the proportion of looks to the model and build AOIs at the start of the 3-s window prior to gesture onset, as well as the maximum or minimum values with corresponding 95% confidence intervals. The *bottom left panel* depicts the relative total dwell time on the face AOI when another person makes a point gesture; ask, verify, or check gesture (labeled ‘ask’); instruct gesture, or no gesture. The bottom right panel depicts the relative total dwell time on the face AOI during episodes that the other person was quiet or spoke. *Grey bars* represent the means across all participants with standard error of the mean. *Orange markers* represent averages for each participant. The *numbers* indicate how many participants contribute to each average. Note that the limits of the vertical axis differ between the bottom two panels

The top panels in Fig. 5 depict gaze to the three AOIs on the table as a function of time to the onset of the other person’s place or remove action for all individuals. Consider the place action first (top left panel). While much less pronounced than the gaze-action coupling and gaze-gesture coupling described above, it seems that there is a peak in gaze to the blocks AOI just prior to the gesture onset (around -0.5 s). After gesture onset (0 to 1 s), the proportion of looks to the model AOI slightly decreases, while the proportion of looks to the build area increases slightly. This might be construed as a person checking the blocks and build areas around another person’s place action to update their internal model of the progress on the model-copying task. For the remove action (top right panel), there is a decrease in the proportion of looks to the model area and a slight increase in the proportion of looks to the build area around and just after the action onset. Similarly, this might be construed as a participant updating their internal model of the current task progression. To quantify these effects, we compare the proportion of looks to the model and build AOIs at the start of the 3-s window to the minimum or maximum value in the -3 to +3 s window. The minimum and maximum values all occurred just around the onset of the other person’s action. For the place action, an approximately 3% increase in the proportion of looks to the blocks AOI was observed prior to the action onset. After the action onset, an approximately 8% decrease in the proportion of looks to the model AOI was observed, combined with an approximate 7% increase in looks to the build AOI. For the remove action, we observed an approximate 18% decrease in the proportion of looks to the model AOI, combined with a 14% increase in looks to the build AOI. Note that the 95% confidence intervals do not, or hardly, overlap.

For the coupling between gaze and another person’s gesture and speech, we observe similar patterns as described above for the gaze-gesture coupling. Again, we consider the total time on the face during the entire gesture or speech episode, as the analyses for timing relative to gesture and speech onset did not reveal noteworthy patterns. The bottom left panel in Fig. 5 shows that the relative total dwell time to the face is very low when the other person makes no gesture, but seems higher when the other person makes ask type of gestures. Again, this is only the case for some, but not all participants (as can be seen from the orange markers). Notably, the relation between the relative total dwell time to the face and another person’s gestures is less pronounced than depicted above for the gaze-gesture coupling. No obvious relation was observed between relative total dwell time to the face of the other person and that person’s episodes of speech (bottom right panel).

**Fig. 6:**
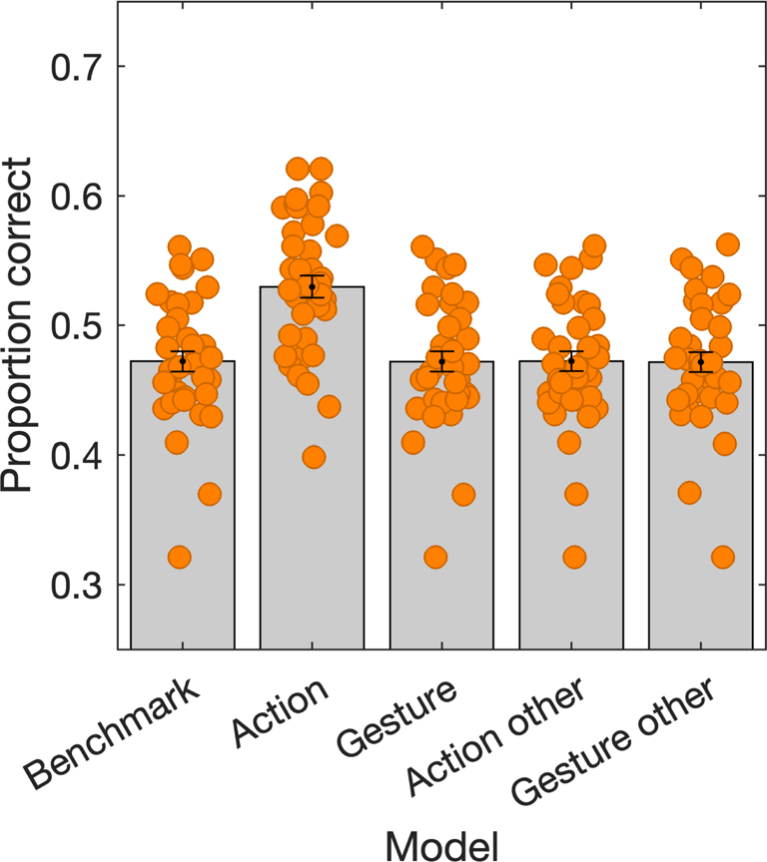
Model performance for inferring gaze behavior from one’s own manual actions and gestures and from the interacting partner’s manual actions and gestures. Performance is expressed as the proportion of correctly labeled gaze samples, i.e., each recorded gaze location in the 60-Hz data for four 60-s trials. *Grey bars* represent the means across all participants with standard error of the mean. *Orange markers* represent values for each participant. Note that the models for speech onsets did not yield any different predictions from the benchmark, i.e., were numerically equivalent to the benchmark, and are therefore not depicted

### Modeling

Figure 6 depicts model performance for inferring gaze behavior from the onsets of one’s own manual actions and gestures, and the interacting partner’s manual actions and gestures. Only the model based on one’s own actions outperforms the benchmark (53% vs. 47%, respectively), which is based on the AOI that is most looked at (i.e., guessing the majority class). Note that the models based on the onsets of one’s own speech and the interacting partner’s speech are not included because they did not alter the prediction relative to the benchmark (i.e., they were numerically equivalent). Thus, knowledge of an upcoming or preceding manual action yields a better inference of gaze behavior compared to the benchmark. Knowledge of an upcoming or preceding gesture, or of the interacting partner’s actions or gestures does not yield better inference of gaze behavior compared to the benchmark.

## Discussion

This study explored gaze behavior for information-uptake and communication in the context of collaborative tasks. We investigated and quantified patterns of (1) within-person gaze-action coupling, i.e., visually guided execution of task steps, (2) within-person gaze-gesture and gaze-speech coupling, and (3) coupling between one person’s gaze and another person’s manual actions, gestures, or speech, during a collaborative Lego Duplo-model copying task from Hessels, Teunisse, Niehorster, Nyström, Benjamins, Senju, Hooge (2023). In addition, we investigated how well gaze behavior could be inferred from one’s own manual actions, gestures, or speech behavior, or that of the person one collaborates with. To the best of our knowledge, this is the first study addressing within-person gaze-action coupling, multimodal communication, and gaze behavior in response to the interacting partner (i.e., exogenous attraction of gaze) simultaneously in the context of one interaction.

Regarding within-person gaze-action coupling, we observed a strong (changes up to 60–70% of looks to the AOIs) relation between the manual action and the gaze location in the world around the action onset, matching previous research (e.g., Ballard, Hayhoe, Pelz ; 1995, Land, Mennie, Rusted ; 1999, Hayhoe ; 2000). Numerically, this conforms very closely in timing and magnitude to similar recent research on gaze-action coupling (see Fig. 3 in Keshava, Nezami, Neumann, Izdebski, Schüler, König ; 2024) indicating the potential generality of looking for grasping actions. However, we did observe slight differences in the timing of gaze-action coupling in comparison to Land, Mennie, Rusted (1999). They concluded that gaze precedes manipulation by approximately 0.5 s (see also Helsen, Elliott, Starkes, Ricker ; 1998, Helsen, Elliott, Starkes, Ricker ; 2000, for research showing that gaze precedes the hands during manual aiming). In our case, gaze to the action-relevant AOIs peaks at 0 s to action onset. However, our action onsets are defined by the movement going into the respective areas, e.g., the grab action starts when the participant initiates the movement into the blocks area, not the moment of manipulation (i.e., grasping the block) as in Land, Mennie, Rusted (1999). We opted for this procedure to ensure that actions could be annotated consistently. Our action starts are therefore earlier in time than by the operationalization of Land, Mennie, Rusted (1999). Thus, these apparent differences in timing are likely due to the exact operationalization. Gaze does indeed precede the manipulation of the building blocks in time for grab and place actions.

Regarding within-person gaze-gesture and gaze-speech coupling, we made three observations. First, we found a relation between gaze to the model, build and block AOIs and the onset of the pointing gesture, specifically with gaze being more likely to be directed to the model AOI just prior to the pointing gesture. Note that the magnitude of this relation was smaller (changes up to 25% of looks to the model AOI) than that observed for the gaze-action coupling described above. Second, we found that overall, throughout the experiment, the face of the other person was hardly ever looked at. However, some participants looked much more at the face of the other while making ask or instruct gestures. Whether this behavior may be related to more general idiosyncrasies observed in gaze to bodies or faces (cf. Peterson and Eckstein ; 2013, Mehoudar, Arizpe, Baker, Yovel ; 2014, Peterson, Lin, Zaun, Kanwisher ; 2016, Hessels, Benjamins, van Doorn, Koenderink, Holleman, Hooge ; 2020a) is an interesting open empirical question. Third, we find no relation between gaze to the face of the other person and one’s episodes of speech, which is in contrast to substantial research on gaze to faces during conversation (e.g., Kendon ; 1967, Kendon , Ho, Foulsham, Kingstone ; 2015, Haensel, Smith, Senju ; 2022). It seems that the patterns of gaze to faces generally observed in face-to-face conversation are overridden completely by the instruction to recreate the models together. Participants are focused on the building process, and need not look at each other while conversing.

Regarding coupling between one person’s gaze and the other person’s actions, gestures, or speech, we made two observations. First, we find a relation between gaze and the remove and place actions of the other person. Gaze was more likely to be directed to the build area just after the other person made a place or remove action there. This may be considered as instances of what Hayhoe and Ballard (2014) describe as “exogenous stimuli that can change the agent’s agenda” (p. R628). However, the magnitude of this relation (changes up to 18% of looks to the AOIs) is much smaller than that of the gaze-action coupling, and also smaller than that of the gaze-gesture coupling described above. Second, we found that some participants looked more at the face of the other while the other person made an ask or instruct gesture, than during the rest of the experiment.

In sum, we find patterns of within-person gaze-action coupling, within-person gaze-gesture coupling, and coupling between one person’s gaze and another person’s manual actions and gestures. The magnitude of these patterns – i.e., the maximum difference in the proportion of looks to one AOI in the -3 to 3 s window around the onset – where largest for gaze-action coupling, followed by gaze-gesture coupling and exogenous attraction of gaze. Hereafter, we tried to infer the gaze location during the collaborative Duplo model-copying task from one’s own actions, gestures, speech or those of the interacting partner. Only knowledge of when one’s own actions took place or would take place led to better inference than a benchmark of guessing the majority class. Note that we also tried decision tree and naive Bayes classification models to infer gaze behavior. These did not yield better performance than those described in this paper.

Why didn’t information about one’s own gestures or that of the other person’s actions or gestures yield better inference than the benchmark, given that we observed statistical relations to the gaze location (see Figs. 4 and 5)? There are at least two reasons why this might be the case. First, there is a strong benchmark already, namely a high probability that the model AOI is fixated (47% of the time). For the relation between gaze location and one’s own pointing gestures, there is a substantial change in the proportion of looks to the model AOI (see top left panel in Fig. 4), yet the majority class remains the same. Thus the inferred gaze label for the time around the pointing gesture will always be the model AOI. For the relation between gaze location and the other person’s place or remove actions, the same principle holds (see top panels in Fig. 5). For the remove action, there is only a very brief change in the majority class. Second, gestures did not occur as often as manual actions (see Table 1), thus making the potential for improvement to the benchmark small.

One might therefore be inclined to conclude that visual routines theory, while not developed specifically for face-to-face collaboration, is the only relevant model in interactions like our collaborative model-copying task. However, there was only a 6% increase in performance for the action model compared to the benchmark, which seems underwhelming. A 6% increase in performance translates to an additional 3.6 s of gaze data (6% of the 60-s trials) that were labeled correctly compared to the benchmark. For the 20 manual actions that occur on average in a 60-s trial (see Table 1), which means an approximately 180 ms per action is additionally correctly inferred. Based on Fig. 3, we might have expected more, e.g., because the likelihood of fixating the blocks area during a grab action exceeds the likelihood of fixating the build or model area for about 1 s. The same holds for the likelihood of fixating the build area during a place action. There may be several reasons for this. First, it may be that there are individual differences in the timing of gaze to the different AOIs in relation to making a manual action. Second, it may be that our annotations of action onsets did not accurately capture the timing of the physical interaction between hand and blocks. Regardless, the gaze location only seems to be predictable just around the onset of the manual action. When no action takes place, individuals may differ substantially in where they look in the world and when (cf. Hessels, Benjamins, van Doorn, Koenderink, Holleman, Hooge ; 2020a, Ghiani, Amelink, Brenner, Hooge, Hessels ; 2024).

One of our aims was to potentially reveal the relative contribution of gaze-action coupling, gaze-gesture and gaze-speech coupling, and exogenous attraction of gaze to gaze behavior in collaborative interaction. Although we observed statistical relations between gaze location and manual actions and gestures, only one’s own manual actions seemed to lead to minimally better inference of gaze location. Note that our models could even look into the future at what action or gesture was upcoming, which is unrealistic for an agent acting in the world, but should yield the best inference performance. What does this mean for the attempt at integrating gaze for information-uptake with gaze for communication into models of face-to-face collaboration? We see at least several implications. First, the relation between speech and gaze to faces that is strong in conversations can be overridden almost completely by the model-copying task (see also Macdonald and Tatler ; 2018). Second, inferring gaze from manual actions seems most realistic for constrained tasks in which different manual actions follow in a quick sequence. Third, gaze-gesture coupling and exogenous attraction of gaze are much less pronounced than gaze-action coupling and they may be highly context-dependent. For example, Andrist, Collier, Gleicher, Mutlu, Shaffer (2015) and Mihoub, Bailly, Wolf, Elisei (2016) also report a relation between gaze location and gestures, speech, or the phases of the interaction. Andrist, Collier, Gleicher, Mutlu, Shaffer (2015) report that gaze location is useful in predicting the phase of the interaction (i.e., making a request vs. completing an instruction), and Mihoub, Bailly, Wolf, Elisei (2016) show that co-verbal actions (gaze and gestures) can be generated from speech and the actions of the other partner. Crucially, these two studies employed an instructor–instructee type of interaction, where actions are completed in a much more sequential fashion between the interactors. In our study, both partners can (re)act simultaneously, which may make it more difficult to annotate or determine the states of the collaboration.

One limitation of our study was that we categorized gaze location in the world into the face and three large AOIs on the table (model area, build area, and blocks area). It may be that gaze location can be predicted at a more fine-grained spatial scale too, particularly in relation to the (non-)verbal communication. For example, consider one participant indicating verbally and by pointing that a particular red block needs to be placed two places to the left. The other person may shift their gaze from where they are looking at the build area to this particular block and where it needs to be placed. However, this is all one AOI in our study. Because of the inaccuracy of the eye trackers, as well as for practical reasons of automating data analyses, it was not possible to conduct a spatially more fine-grained analysis of the gaze data. In order to conduct such analyses, one would need a moment-to-moment understanding of the spoken language and its potential referents (cf. Hanna and Brennan ; 2007, Macdonald and Tatler ; 2013) as well as eye trackers that are accurate and precise enough to deliver gaze data at the spatial scale at which these referents occur. Looking forward, we also see the utility of modeling when a clear relation between gaze behavior and other (non-)verbal behavior is to be expected. If, for example, a human deviates from this, we might ask what information they are missing from the interaction. Such models may also be relevant for e.g., the development of virtual agents and (social) robotics, or for application in human–robot interaction. Secondly, it should be noted that we restricted ourselves to the analysis of gaze behavior of individuals in relation to their own actions, gestures, and speech episodes, or those of the person they interacted with. Future work could also consider modeling the state of the interaction at the level of the dyad (i.e., concurrent gaze locations, or combined gesture or speech behaviors). The reason that we preferred the individual perspective was that previous research on visual routines, multimodal communication, and the exogenous attraction of gaze was also at the level of the individual. Our contribution is specifically in the combination of these perspectives in the context of collaborative interaction, and quantifying the relative magnitude of gaze-action coupling, gaze-gesture coupling, and exogenous attraction of gaze in the context of the same collaborative interaction. We welcome future work from a more interactive perspective (cf. Pickering and Garrod ; 2004, Hutchins ; 2010, Fusaroli and Tylén ; 2016, Dingemanse, Liesenfeld, Rasenberg, Albert, Ameka, Birhane,... Wiltschko ; 2023).

To conclude, we show that patterns of within-person gaze-action coupling, within-person gaze-gesture coupling, and coupling between one person’s gaze and another person’s manual actions and gestures can all be observed in the context of the same dyadic collaborative task. However, inferring gaze location only exceeded a benchmark for a model including one’s own manual actions. The improvement was minimal and less than what may have been expected based on visual routines theory. We suggest that inferring gaze location may be most effective for constrained tasks in which different manual actions follow in a quick sequence, while gaze-gesture and gaze-speech coupling may be stronger in unconstrained conversational settings.

## Data Availability

Data files and example videos are available at https://osf.io/2q6f4/.

## Declarations

### Open Practices Statement

Data files and example videos are available at https://osf.io/2q6f4/. The experiment and analyses were not preregistered.
